# Patient-centricity in digital measure development: co-evolution of best practice and regulatory guidance

**DOI:** 10.1038/s41746-024-01110-y

**Published:** 2024-05-16

**Authors:** Suvekshya Aryal, Jennifer M. Blankenship, Shelby L. Bachman, Soohyun Hwang, Yaya Zhai, Jennifer C. Richards, Ieuan Clay, Kate Lyden

**Affiliations:** VivoSense Inc, Newport Coast, CA USA

**Keywords:** Drug regulation, Drug development

## Abstract

Digital health technologies (DHTs) have the potential to modernize drug development and clinical trial operations by remotely, passively, and continuously collecting ecologically valid evidence that is meaningful to patients’ lived experiences. Such evidence holds potential for all drug development stakeholders, including regulatory agencies, as it will help create a stronger evidentiary link between approval of new therapeutics and the ultimate aim of improving patient lives. However, only a very small number of novel digital measures have matured from exploratory usage into regulatory qualification or efficacy endpoints. This shows that despite the clear potential, actually gaining regulatory agreement that a new measure is both fit-for-purpose and delivers value remains a serious challenge. One of the key stumbling blocks for developers has been the requirement to demonstrate that a digital measure is meaningful to patients. This viewpoint aims to examine the co-evolution of regulatory guidance in the United States (U.S.) and best practice for integration of DHTs into the development of clinical outcome assessments. Contextualizing guidance on meaningfulness within the larger shift towards a patient-centric drug development approach, this paper reviews the U.S. Food and Drug Administration (FDA) guidance and existing literature surrounding the development of meaningful digital measures and patient engagement, including the recent examples of rejections by the FDA that further emphasize patient-centricity in digital measures. Finally, this paper highlights remaining hurdles and provides insights into the established frameworks for development and adoption of digital measures in clinical research.

## Introduction

Digital health technologies (DHTs) offer the ability to capture data remotely, continuously, and with low patient burden^1–3^. As such, DHTs are paving the way for the development of a new class of clinical outcome assessments (COAs) that reflect how patients feel, function, and survive in real-world environments^4^. Many believe the evidence generated from DHTs provides an opportunity for a more holistic and direct understanding of the patient experience^2,5^, and consequently, there has been a significant investment into developing new DHT-derived outcome measures^6^. Initial efforts were technology-driven and centered around expanding the limits of what can be measured^1,7^. However, following the overall trend in the industry for a more patient-centric approach to drug development, there is an increased focus on understanding what should be measured with DHTs^1,2,8–12^.

The US Food and Drug Administration (FDA) has progressively evolved their guidance and exerted pressure on drug developers to move towards a more patient-focused approach, encouraging the systematic incorporation of the patient voice into all aspects of drug development and evaluation^13^. Over the past decade, there has been an increasing emphasis on engaging patients in protocol development, endpoint selection, and the development of fit-for-purpose evidence generation tools^12^. Both the 2009 Patient-reported Outcome (PRO) guidance for industry^14^ and the 2012 Patient-Focused Drug Development (PFDD) initiative^12^ provide roadmaps on how to incorporate the patient experience into medical product development. The most recently released four-part guidance series further emphasizes this by providing stepwise recommendations on how to collect and submit patient experience data for regulatory decision making. Collectively, the goal of these initiatives is to develop interventions that induce a treatment benefit that is considered meaningful to patients.

This viewpoint aims to illustrate how the recent FDA PFDD guidance has shaped digital measure development with a particular emphasis on generating evidence that DHT-derived COAs are meaningful to patients. This paper discusses various methodologies available to establish the meaningfulness of digital measures required to facilitate regulatory endorsement, and highlights the progress made, hurdles that remain, and future directions needed for the successful adoption and implementation of digital measures.

### Shaping digital measure development: the role of regulatory guidance

The scientific, financial, and operational advantages of digital measures in drug development have been extensively discussed^6,15,16^, however, despite this, adoption has been limited to date. Some of the resistance from sponsors is due to the uncertainty that endpoints derived from DHTs will be of value in discussions with regulators. Given the importance of PFDD at the FDA, establishing clear rationales of meaningfulness of digital measures provides the best opportunity for regulatory qualification, which in turn paves the way for broad utilization in future drug development trials. Digital measures capture aspects of life in real-world environments, and compelling cases can be made that outcomes derived from DHTs will be inherently meaningful to patients, yet robust evidence is required to support those arguments. Recent guidance describes what is required to justify the seemingly simple statement that a new digital measure is “patient-centric”.

### Understanding the FDA guidance on driving patient-centric digital measure development

The FDA’s recent four-part guidance on PFDD significantly expands their position on patient engagement in drug development^17^. This guidance series is applicable to all COA categories (i.e., PROs, clinician-reported outcomes, observer-reported outcomes, and performance outcomes), and outcomes derived from DHTs. Part 1^18^ and part 4^19^ of the series primarily address comprehensive and representative input from patients and caregivers for regulatory decision making. Part 2^20^ focuses on the methods and best practices for gathering patient input on what is important to them in disease and treatment, including a focus on psychometric testing. Part 3^21^ provides a general roadmap for developing patient-focused outcome measurement and supporting a COA as fit-for-purpose. It also provides recommendations for evaluating COAs based on their construct and content validity, as well as reliability and responsiveness to change. While the FDA has consistently required evidence to support the claim that COAs are selected based on patient-centric concepts^22^, this recent guidance series emphasizes the importance of adopting robust methodologies to generate such evidence.

Digital measure developers have started adopting this guidance to initiate regulatory submissions amidst discussion of a dedicated pathway for deploying digital measures as drug development tools (DDTs)^23^.

### Evolving FDA expectations on incorporating meaningfulness in digital measure development

In the history of the FDA DDT-COA qualification program^4^, which evaluates whether COAs are a well-defined and meaningful assessment of how patients feel, function, and survive in specified contexts of use, 9 letter of intents (LOIs) for DHT-derived COAs have been submitted for qualification^24^. The earliest submission of an LOI accepted into the qualification program was reported in 2018 for the assessment of physical activity via accelerometry^25^. In total, 6 LOIs for DHT-derived COAs have been accepted into the program; however, it has been 4 years since the last LOI was accepted by the FDA. The most common reason for LOI rejection is a lack of meaningfulness of the measure to patients, which is pushing developers to align with FDA’s evolving expectations, particularly for the projects that were planned or submitted years before the recent guidance was released.

For example, the FDA rejected an LOI for a DHT-derived COA assessing motor symptom severity using a finger tapping test on a smartphone, for use in Parkinson’s disease^26^. While a case could be made that maintaining fine motor skills is important for patients with Parkinson’s disease, in the rejection letter FDA was concerned about the meaningfulness of the act of tapping repeatedly on a screen to patients^26^.

Similarly, the FDA rejected an LOI for a COA reflecting aspects of gait abnormality in Huntington’s Disease because the advanced gait assessment proposed was not inherently easy to understand. Further, as indicated in the decision letter, there was a general lack of clarity on whether changes in the proposed gait parameters were meaningful to patients and if changes in gait parameters would reflect changes in everyday functioning^27^. The FDA’s response was consistent in its most recent rejection of an LOI for an actigraphy-derived COA that reflected aspects of functional mobility such as gait, balance and coordination for use in neurodegenerative diseases^28^. The reviewers expressed concerns as to whether the measures are relevant and important to functional mobility and whether the gait kinematic parameters suggested in the conceptual model would be meaningful to patients. To note, there has been an uptake of DHTs in the neurological field, signaling early integration in clinical research, particularly in Parkinson’s disease^29^. Evidence of patient-centric development will inform success in regulatory approval and wider adoption, but the approach needs to be tailored to the patient population. For example, in Alzheimer’s disease, patients find difficulties articulating their lived experiences due to cognitive decline, and so the development effort may require more nuance.

The rejections in the FDA COA qualification program indicate a shift in the rigor of evidence required to demonstrate that digital measures are meaningful to patients^30^. A separate guidance, issued for remote data acquisition using DHTs in clinical trials^31^, outlines that evidence is needed to demonstrate that a DHT is usable and acceptable by patients, accurately measures the outcomes it claims to measure, and that the derived outcomes are clinically relevant to the specific population (i.e. conforms to evidentiary standards detailed in the V3 framework^32^). Although evidence collection along this continuum is critical for DHT-derived COAs and biomarkers to be accepted or qualified by regulators, FDA’s recent guidance series related to meaningfulness of the measures captured by the DHTs is at the forefront. Alignment across stakeholders around the V3 framework has catalyzed innovation, leading to adaptations of established methodologies and concepts from the “traditional” biomarker field^33^. Current regulatory guidance is now prompting a similar cross-pollination, with digital measure developers increasingly turning to established practices from fields like psychometrics research, product development and user experience, in order to robustly demonstrate that the chosen measurement concepts are meaningful to patients. The FDA also offers various channels of early engagement for submissions, for example, through the Critical Path Innovation Meeting (CPIM) for COAs^34^ and pre-LOI program for biomarkers^35^. This encourages a dialog between researchers, developers and regulators in the pre-competitive or pre-submission stage.

### Establishing meaningful aspects of health in practice

#### Engaging with patients to determine meaningful aspects of health

The Digital Measures that Matter framework^1^ provides a hierarchical model for establishing meaningfulness. First, patients’ experiences are mapped to high-level Meaningful Aspects of Health (MAH). MAH are then linked to broader and specific measurable concepts of interest, and finally to specific outcomes and endpoints. The framework helps to understand meaningful evidence as something rooted in MAH and avoid “technological determinism”, i.e. defining a patient’s lived experience by what can be measured with a technology instead of first considering what should be measured. Similarly, the PFDD guidance emphasizes using robust methods and psychometric analysis to gather patients’ experience and input on important aspects of life that should be assessed in the disease and its treatment^20,21^. The following sections present a brief recap of the key methodologies available to determine meaningful measurement concepts. The best method to gather patient input must be determined on a case-by-case basis and is dependent on the specific needs and context of the research in question.

The FDA encourages facilitated discussions with patients and caregivers to incorporate patient voice through the established model of FDA-, or externally-led PFDD meetings that organizations can adopt for collaborative meetings with patients and key stakeholders in any specific disease area^36^. Consensus on high-level MAH is often gathered in these meetings, catalyzing further research that can then focus on determining specific measurable concepts or outcomes linked to identified MAH. Qualitative research methods, such as one-on-one interviews and focus group discussions, are recommended to gain a deeper understanding of individual or group experiences. Quantitative methods such as surveys can also be useful for gathering patient input, but considerations should be taken to develop clear, concise and relevant questions and pilot with small groups of participants before launching larger studies. Mixed-methods approaches that integrate quantitative and qualitative approaches can provide a comprehensive understanding of patient perspectives and can help understand the generalizability of any observations.

These methods can provide foundational evidence to support the meaningfulness of novel DHT-derived COAs. The examples discussed below and in Table 1 showcase how sponsors and researchers are using the Measures that Matter framework and the roadmap developed by the FDA to gather patient input for COA development.

**Table. 1:** Identifying meaningful aspects of health in digital measure development: key examples

Therapeutic Area	Digital Measure	Research Team	Methods for Identifying MAH	Regulatory Success
Atopic Dermatitis	Nocturnal scratch	Digital Medicine society (DiMe) multi-stakeholder collaborative	- MAH were established through concept-elicitation interviews with 49 patients comprising adults, children and caregivers, which informed a conceptual model for scratching as a concept of interest^37^. - In a survey, experience and burden of nocturnal scratch was further explored, including their preferences on treatment options and their willingness to use DHTs to measure nocturnal scratch^37^.	LOI accepted by FDA (in a separate submission for scratch sensor in atopic dermatitis)^58^
Idiopathic Pulmonary Fibrosis (IPF)	Moderate-to-vigorous physical activity (MVPA)	Bellerophon Pharmaceutical	- Preliminary evidence of MAH were generated with the help of the Voice of Patient report from an FDA-led PFDD meeting including 60 IPF patients^16^. - Patients expressed that IPF limits their ability to perform activities of daily living, particularly those which require high levels of exertion like walking, climbing stairs, and exercise^59^.	Endpoint accepted by FDA in a phase 3 IPF clinical trial
Duchenne Muscular Dystrophy (DMD)	Stride velocity 95^th^ centile (SV95C)	ActiMyo® research collaborative	- MAH were established through qualitative investigation that included discussions with key clinical experts and stakeholders from private and public organizations and a survey of patients and caregivers that gathered in-depth feedback on the meaningfulness of ambulation in DMD and acceptability of wearable devices in this context^40^.	LOI accepted by FDA^39^; and endpoint qualified by EMA in DMD clinical trials^40^

#### Obtaining diverse patient input to establish meaningfulness: key examples

In an example of patient-centric development, a mixed-methods study was conducted to understand MAH in atopic dermatitis^37^. The results found that scratch was the most burdensome and the most bothersome symptom that kept patients awake at night. The patients’ treatment considerations included relief from nocturnal scratch and adopting technologies that would measure scratch. This evidence is key for development and discovery of novel products and interventions that improve the lives of many individuals living with atopic dermatitis.

In another example, findings from an FDA-led PFDD meeting were used to identify MAH^16^ in idiopathic pulmonary fibrosis (IPF). The Voice of the Patient report provided the justification that higher-intensity physical activity is meaningful to patients with IPF. As a result, moderate-to-vigorous physical activity derived from a wrist actigraphy sensor was incorporated as a primary endpoint in a Phase 3 IPF trial^38^. This serves as a key example of how existing sources of evidence should be leveraged to gather patient input whenever possible.

Additionally, the development of stride velocity 95^th^ centile (SV95C) in Duchenne Muscular Dystrophy (DMD)^39^, with an accepted LOI and endpoint qualified by EMA, established MAH through qualitative investigation in clinical experts, and patients and caregivers^40^. Evidence from these efforts indicated that patients and their caregivers view ambulatory function as key to their independence, want to maintain or improve it through treatment, and consider its real-world measurement important. (**See** Table 1).

#### Co-creating a conceptual framework with patients

Patient involvement in digital measure development is crucial beyond the initial step of establishing MAH. In the subsequent step of developing a conceptual framework, concepts identified as MAH are linked to measurable outcomes and then mapped to specific endpoints^21^. At this stage, patient input is important for ensuring that identified concepts and outcomes resonate with their lived experiences.

Such co-creation is the hallmark of Mobilise-D’s development effort. The consortium is developing digital measures of mobility in various conditions such as Parkinson’s disease, multiple sclerosis, chronic obstructive pulmonary disease, hip fracture, heart failure, frailty and sarcopenia^41^. Their work includes an exhaustive literature review of qualitative evidence of meaningfulness of walking. Patients were directly involved in the interpretation of the findings, where they helped identify several concepts of walking experience that are universally meaningful^42,43^. This level of patient engagement confirms that development efforts are rooted in patient experience.

### Engaging with patients to evaluate user experiences and deliver value with DHTs

Meaningfulness in defining the measured concept is, of course, a foundational step, but this work is inconsequential if the implementation of the digital measure is not thoughtful^6^. Engaging patients provides a path to inform what evidence needs to be considered, how a measure will be implemented, and whether direct value for patients will be delivered. Therefore, once the conceptual framework supporting a digital measure is established, evidence of usability and acceptability of the relevant technology should be generated, beyond the required verification and validation^32^. As such, meaningfulness can be enhanced by ensuring that a measure that is rooted in patient experience is also acceptably captured by DHTs based on the user experience^31^. Equally, thinking of the value that can be directly delivered to patients, for example through the return of a summary of health data or trial results, opens further opportunities for engagement.

#### Establishing DHT acceptability and feasibility through real-world testing

There are various factors to consider while user testing DHTs, such as technical features, ease and comfort of use, interference with daily life, and the perceived benefit of use in the real-world^43,44^. Engaging with patients who have used and tested a given DHT in their lived environment is critical to generating such evidence. In a notable example, IDEA-FAST, another IMI-funded research consortium aiming to develop digital measures of sleep, fatigue, and activities of daily living in neurodegenerative and immune-mediated inflammatory diseases, has ongoing work to directly evaluate feasibility of using multiple sensing devices^45^. Researchers are using mixed-methods approaches to evaluate user experience, acceptability, and compliance. Patients’ daily reports are collected through questionnaires and daily diary, and objective measures of sensor wear time and compliance are collected directly from the DHTs^46^. Such data are also critical to evaluate whether DHTs are feasible for continuous and reliable data collection in the real world^44^.

To ensure representativeness of a DHT’s acceptability and feasibility, it is crucial that real-world device testing is performed in individuals from diverse socio-demographic backgrounds and disease severities. As DHTs pave the path for decentralized trials, gathering a broad spectrum of experiences becomes a priority in order to achieve inclusivity and representation in clinical trials^47^. Moreover, understanding the patient’s journey requires recognizing that distinct “personas” and preferences are dynamic and differ significantly among patient groups. For example, while a given wearable technology might be well received by patients with mild Alzheimer’s disease, its acceptability may be different for those with moderate and severe forms of the disease. Similarly, for cancer patients undergoing intensive treatment, the tolerance for wearable sensors may be diminished during treatment compared to other times.

#### Returning DHT-derived data back to the patients

Return of summarized health data to participants, especially those collected using DHTs, has recently been recognized as preferred by patients^48^ and known to help motivate patients and encourage communications with clinicians^49^. This serves as an opportunity for continuous patient engagement during the digital measure development process. It is also one way to ensure that meaningfulness is retained when progressing from the establishment of MAH and conceptual framework to digital measure deployment. In IDEA-FAST’s feasibility study utilizing multiple sensing devices (measuring physical activity, cognition and other health metrics), a summary of health data was provided back to patients at different time points during the study. To understand the importance of returning data and the best mechanisms to do so, the study explored patients’ perceived benefits of access to data, as well as use and interpretability of the data^50^. The preliminary findings suggested that patients reported using the information to monitor their health and performance, and felt confident about their adherence to the devices, which also fostered communications with clinicians. Some patients had difficulties interpreting the data, and some were confused about missing data. While the research on this front is still new, providing patients with access to their post-study data can be valuable to continue engagement at the final stage of digital measure deployment. As such, innovations on this front, for returning accessible and interpretable data, such as through mobile app features or summary reports, may be a worthwhile investment.

Traditionally, in clinical trials such return of data or results is uncommon. The return of DHT-derived data as discussed here is in line with the patient engagement efforts crucial for generating the evidence required for identifying and developing digital measures that are ultimately integrated in the clinical trials. Nevertheless, this must be approached with caution, and depending on the type of study there may be ethical and legal implications as well as errors or biases impacting patient behavior and the data thereafter^51^. Key considerations here include clear communication with patients (in informed consent), and proper documentation of plans for handling incidental findings and ways of mitigating risks (in IRB application)^52^.

### Remaining hurdles for digital measure development and adoption

The past 10 years of FDA PFDD guidance have culminated in a deepening focus on patient engagement throughout the process of DDT-COA development. The patterns observed in the FDA COA qualification submissions reveal an increased emphasis on meaningfulness to patients, underscoring the importance, for acceptance and qualification by regulators, of developing digital measures that resonate with patients’ lived experiences in disease or treatment. As such, the regulatory guidance requires a continuous co-evolution with the digital measure field.

**Fig. 1:**
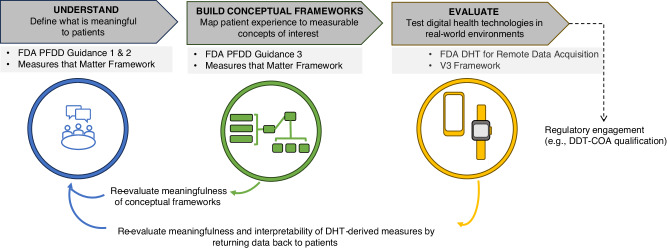
Roadmap for co-creating meaningful digital measures with patients. Guidance from FDA^17,31^ and others^1,32^ emphasizes the importance of developing measures that matter to patients. The established process of developing digital measures that matter to patients (upper dark gray boxes) involves intermittent engagement with patients to define what is meaningful and determine the feasibility of capturing measures with DHTs (applicable guidance noted in light sgrey boxes). Several consortium projects have put this approach into practice; these include DiMe^37^, Mobilise-D^60^, and IDEA-FAST^61^. To move towards co-creating digital measures with patients, providing value to patients throughout the process of development is a necessary next step. As depicted in the lower section of the figure, this approach ensures the measures developed remain rooted in the patient voice and offer opportunities to iteratively refine the foundational evidence supporting such measures. DDT-COA Drug Development Tool-Clinical Outcome Assessment, DHT Digital Health Technologies, DiMe The Digital Medicine society, FDA Food and Drug Administration, PFDD Patient-Focused Drug Development.

The FDA showcases commitment to building internal infrastructure and expertise to continue to advance the field. The future looks promising as both developers and regulators realize the significant benefits of PFDD in improving patient outcomes with increased efficiency, expedited regulatory decisions, and enhanced stakeholder engagement^6,15,41^. Figure 1 summarizes a roadmap for frequent patient engagement throughout the process of digital measure development. The roadmap also suggests an iterative process of communicating the results back to the patients and refining the development approach so that the meaningfulness is retained throughout.

Despite progress, challenges persist. First, patient experiences can be highly heterogeneous making it difficult to identify a single aspect of health that is meaningful and generalizable across all patients. Further, the ways in which a single aspect of health manifests across patients may also differ. For example, the fear of losing independence, while common across different phases of a patient journey, may translate to different measurement concepts depending on multiple factors including disease severity, culture, or other socioeconomic variables.

Secondly, even with detailed regulatory guidance on developing meaningful measures, there is no straightforward or standard path to successful development of a conceptual model. Without a clear roadmap for generating a specific concept of interest from the established MAH, qualification of DHT-derived COAs is challenging. Further, challenges in defining clinical significance remain due to the nature of digital measures and variability across patients and to lack or absence of associated anchors. There are a few examples of successful development in this case, such as the SV95C in DMD^40^, and the FDA’s guidance on PRO (and other COA) development can be utilized to understand methodologies in this context^19^. Still, there is a need for further guidance on these issues.

Furthermore, challenges lie in the classification of digital measures as COAs vs biomarkers in drug development^23^. As many behavioral digital measures are DHT-derived, they occupy a gray area between directly measuring “feel and function” (which would be characteristic of a COA), and a precisely defined, yet relatively abstract, measurement concept (which would be characteristic of a biomarker)^23^. Clarity from regulators will be required to understand whether individual measures are more suitable as COAs or biomarkers going forward.

Finally, there is a need to facilitate adoption internationally. In Europe, EMA has also released its own guidance on DHT-based methodologies to support medical products^53,54^. The EMA database points to various developments in this space. At the forefront is the recent qualification of SV95C as a primary endpoint in DMD clinical trials^15,40^. Additionally, Mobilise-D and IDEA-FAST have engaged the EMA for qualification advice on their digital measure development studies^55–57^. The forthcoming regulatory decisions may also provide valuable insights into alignment or divergence in approval mechanisms and approaches of EMA and FDA. Outside of EMA and FDA, countries such as the UK, Canada, and Japan, as well as international organizations such as WHO, have released guidance for DHTs and real-world data, but specific developments around qualification of measures have yet to be reported.

#### Outlook

The co-evolution of best practice, regulatory expectations, and guidance will progress as more examples of DHT-derived COAs emerge. Furthermore, key frameworks will be implemented and refined, and methodologies from other fields will be adapted in order to fulfill evidentiary requirements. More active engagement and dialog between developers and regulators will be essential to fuel the co-evolution. Thus, there is a need for more developers to take the step of engaging with regulators for qualification and acceptance, and regulators to further clarify and enable digital measure LOIs to be submitted. Engaging interdisciplinary stakeholders is also critical. Scientific and clinical experts play an important role as subject matter experts or principal investigators and support all aspects of the design and implementation. Along with other key stakeholders like the payers, they are vital for advocating for the utility of DHTs. Further, payers are also crucially positioned to improve patient care by establishing reimbursement policies around DHTs and providing wealth of data to facilitate real-world evidence and health economics and outcomes research.

In conclusion, while there are remaining hurdles to the successful development, qualification, and widespread adoption of digital measures, the path forward, driven by co-creation and stakeholder collaboration, holds the promise of a transformed and more patient-centric future in drug development. Ultimately, digital measures that matter to patients and that deliver value to them will prove to be beneficial to all stakeholders.
